# PD-L1 expression and correlation with outcome in muscle-invasive and metastatic urothelial carcinoma: review and critical discussion

**DOI:** 10.3389/fonc.2024.1427452

**Published:** 2024-08-30

**Authors:** Tämer El Saadany, Anja Lorch, Matthew I. Milowsky, Ursula Maria Vogl, Richard Cathomas

**Affiliations:** ^1^ Division of Oncology/Hematology, Cantonal Hospital Graubünden, Chur, Switzerland; ^2^ Department of Medical Oncology and Hematology, University Hospital Zürich, Zürich, Switzerland; ^3^ Lineberger Comprehensive Cancer Center, University of North Carolina at Chapel Hill, Chapel Hill, NC, United States; ^4^ IOSI (Oncology Institute of Southern Switzerland), Ente Ospedaliero Cantonale (EOC), Bellinzona, Switzerland

**Keywords:** PD-L1, regulatory, urothelial carcinoma, adjuvant, metastatic, immunotherapy

## Abstract

Immunotherapy with checkpoint inhibitors including atezolizumab, pembrolizumab and nivolumab has become an essential pillar in the management of muscle invasive and metastatic urothelial carcinoma. The field has evolved quickly in the past few years and several early beliefs have recently been upended. One such belief relates to the predictive value of PD-L1 expression based on immunohistochemistry. Nevertheless, requirements for PD-L1 expression from regulatory bodies still restrict the use of checkpoint inhibitors in urothelial carcinoma. This article provides a critical review of the available data from the registration trials on which the current regulations have been based with the conclusion that a review of the current approval status incorporating PD-1 expression is warranted.

## Introduction

Immune checkpoint blockade (ICB) of the PD-1/PD-L1 (programmed death protein 1/programmed death-ligand 1) signaling pathway, have fundamentally changed the treatment of advanced urothelial carcinoma (UC). Immunohistochemical measurement of PD-L1 expression has been found to be a reliable predictive biomarker in some malignant diseases such as non-small cell lung cancer. Based on initial results published in 2014 (1), it was suggested that this might also be the case for UC. The first evidence, that PD-L1 expression might be an unreliable biomarker in UC derived from the results of the phase 3 trials in second line metastatic UC (mUC), IMvigor211 and Keynote 045 (2, 3).

This literature review aims to evaluate the key findings from registration trials in muscle invasive and metastatic UC, to delineate a clearer understanding of the role of PD-L1 expression on the efficacy of ICB therapy, thereby setting the stage for abandoning the use of PD-L1 immunohistochemistry for clinical decision-making in this disease setting.

## Methods

This literature review was conducted by performing a comprehensive search of PubMed and abstracts from major oncology conferences (ASCO, ASCO GU, ESMO) up to February 2024, related to the use of ICB therapies for the treatment of muscle-invasive or metastatic UC. The search focused exclusively on those pivotal studies that have been used by regulatory bodies for drug approval.

Studies were selected based on their direct relevance to the approval of ICB therapies in the treatment of UC, thus focusing on high-quality evidence that has contributed to the current standard of care for muscle-invasive or metastatic disease. A detailed analysis of the identified studies was conducted, assessing key outcomes including overall survival (OS) and disease-free survival (DFS) in the intention-to-treat (ITT) population and in different PD-L1 expression-based subgroups.

The review also considered the regulatory decisions influenced by these trials, noting differences in approval statuses and indications between the United States of America (Food and Drug Administration [FDA]) and the European Union (European Medicines Agency [EMA]).

## Results

**Figure 1:**
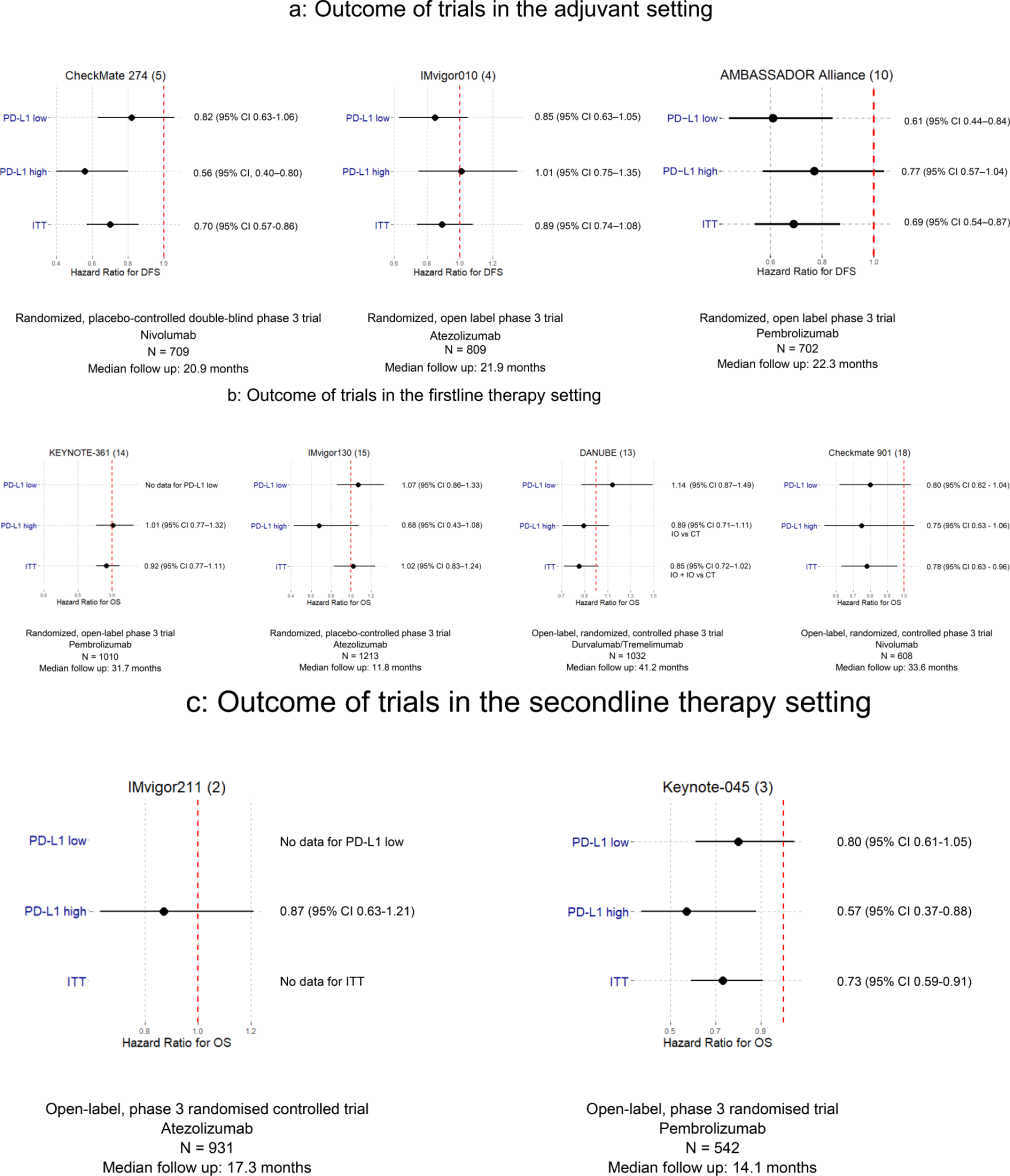
**(A)** DFS comparison according to PD-L1 status – adjuvant trials. **(B)** OS comparison according to PD-L1 status – first line trials. **(C)** OS comparison according to PD-L1 status – second line trials.

The results of the different studies and PD-L1 expression-based subgroups are summarized in **Figures 1A–C**. In addition, **Table 1** informs about the different PD-L1 testing assays and score calculations.

**Table 1:** Different PD-L1 testing assays and score calculations.

Overview of PD-L1 positivity definitions and assays used		
Checkpoint inhibitor	PD-L1 positivity defined as	PD-L1 analysis platform
Atezolizumab	PD-L1 expression of immune cells (IC) IC0 (<1%), IC1 (≥1% and <5%), IC2/3 (≥5%)	VENTANA SP142 IHC assay
Durvalumab	PD-L1 staining of ≥ 25% of TC or ≥ 25% of IC (if >1% of tumor area contained immune cells)	VENTANA SP263 IHC assay
Nivolumab	PD-L1 Expression Level of ≥1% TC or PD-L1 CPS ≥1	DAKO 28-8 pharmDx IHC assay
Pembrolizumab (KEYNOTE)	PD-L1 CPS ≥ 10	DAKO 28-8 pharmDx IHC assay
Pembrolizumab (AMBASSADOR)	PD-L1 CPS ≥ 10	DAKO 22C3 pharmDx IHC assay
Avelumab (JAVELIN Bladder 100)	PD-L1 staining of ≥ 25% of TC, ≥ 25% of IC (if >1% of tumor area contained immune cells) or 100% of IC (if ≤ 1% of tumor area contained IC)	VENTANA PD-L1 (SP263) Assay

CPS, combined positivity score (calculated as the number of PD-L1 positive tumor and immune cells (lymphocytes and macrophages) divided by the total number of viable tumor cells in the evaluable tumor area multiplied by 100); IC, tumor-infiltrating immune cell; IO, immunotherapy; TC, tumor cell.

### Adjuvant setting

Three trials investigated the benefit of ICB in the adjuvant treatment of resected muscle-invasive UC. The IMvigor010 study involving atezolizumab achieved no positive DFS outcome regardless of PD-L1 status (4), whereas the CheckMate 274 trial with nivolumab reported positive DFS results across all patients analyzed (ITT) (5). Notably, patients with positive PD-L1 status exhibited a more favorable hazard ratio and in a subgroup analysis, patients with a PD-L1 expression level of less than 1% in tumor cells did not experience a significant therapeutic benefit (5). A subsequent *post-hoc* analysis, which applied a different calculation for PD-L1 positivity using the combined positivity score (CPS) instead of solely PD-L1 expression on tumor cells, identified 89% of all trial participants as PD-L1 positive, revealing a significant DFS benefit for this group (6). This specific analysis demonstrates that PD-L1 expression measurements can be tailored to fit different arguments and lastly renders them futile. Interestingly in this case, while the FDA approved nivolumab regardless of PD-L1 status (7), the EMA and Swissmedic granted approval only for cases demonstrating PD-L1 positivity of ≥1% on tumor cells (8, 9).

The recently presented AMBASSADOR trial demonstrated a significantly improved DFS for adjuvant treatment with pembrolizumab in the ITT population. Interestingly, a subgroup analysis showed no significant DFS benefit in the PD-L1 high group (CPS ≥ 10%) whereas DFS was significantly improved in the PD-L1 low population (CPS <10%) (10). These results suggest that PD-L1 expression is probably a prognostic but not a predictive factor.

### First line therapy

In the first-line treatment of patients with metastatic UC, the outcomes of ICB therapy have varied significantly in PD-L1-based subgroups. A phase 2 trial with single agent pembrolizumab revealed an advantage for patients exhibiting high PD-L1 expression with longer OS compared to those with lower PD-L1 levels (11). Conversely, in a similar trial, single agent atezolizumab showed that patients with low PD-L1 expression (<5% of immune cells) appeared to benefit more than those with higher PD-L1 expression (≥5% of immune cells) (12).

However, in two major trials (KEYNOTE-361, IMvigor130), ICB therapy did not demonstrate OS superiority over standard chemotherapy, including both ITT populations and PD-1 positive subgroups (13–15). This also applied to the ICB combination of durvaluamb plus tremelimumab (DANUBE) (13). Moreover, a subgroup analysis of cisplatin-ineligible patients from the Keynote 361 study demonstrated no OS benefit for single agent pembrolizumab vs standard carboplatin/gemcitabine (14, 16). Maintenance therapy with avelumab showed a significant OS benefit (JAVELIN Bladder 100). In this study, the treatment was given after a response to previous chemotherapy. Patients with high PD-L1 expression were identical to the corresponding ITT group regarding the OS while there was no OS benefit in patients with low PD-L1 expression (17). Recently the results of the Checkmate 901 trial were reported; an OS benefit was demonstrated for patients treated with nivolumab and cisplatin/gemcitabine chemotherapy regardless of the PD-L1 status (18).

### Second line therapy

In the second line setting, IMvigor211 failed to demonstrate an OS benefit in PD-L1 selected patients, whereas Keynote 045 demonstrated improved OS in the ITT including high and low PD-L1 expression (2, 19).

## Discussion

This review of the registration trials reveals no consistent association between PD-L1 expression and benefit from ICB. There is likely not a single reason with many potential explanations at play. For one, there is significant variability related to the testing performed including antibody clone, scoring system and threshold of positivity. As outlined in **Table 1** several different antibodies are used in test kits, different cells are taken into account (tumor cells, immune cells) resulting in different scores (TPS, CPS, IC) with changing thresholds and finally resulting in the impossibility to compare anything. In addition, prior therapies may alter the expression of PD-L1over time as compared to baseline (20). There also may be discordance in PD-L1 expression between the primary tumor and metastatic lesions temporally and spatially (21). This means, that there is intratumoral heterogeneity within the primary tumor as well as within the metastases rendering any result very difficult to interpret. Moreover, so far unknown differences between ICB drugs might lead to different results in predictive ability of PD-L1.

When examining across all studies, the value of PD-L1 IHC in predicting benefit from ICB is uncertain and it is in fact impossible to understand the value of PD-L1 in UC. For example, it is striking that the predictive value of PD-L1 status in the first line setting in phase 2 single-arm studies (in cisplatin-ineligible patients) and in phase 3 randomized studies turns out to be entirely different with the same drugs (atezolizumab and pembrolizumab) and the same testing kit used in both instances. The same effect is seen in the adjuvant trials and in the second line studies. All of these seemingly contradictory results suggest that PD-L1 expression measured by immunohistochemistry may have prognostic but not predictive value.

The regulatory agencies and the manufacturers have only in part reacted to the updated and contradictory findings with regards to PD-L1 status and outcome in patients with muscle-invasive and metastatic UC. The FDA changed the label to omit requirement for PD-L1 and now has restricted the use of pembrolizumab in the first line metastatic setting to any platinum (cisplatin and carboplatin)-ineligible patients regardless of PD-L1 status (22). In contrast, atezolizumab was approved by FDA for first line therapy in cisplatin-ineligible patients with PD-L1 tumor proportion score of at least 5% or independent of PD-L1 expression for any platinum-ineligible patients but the indication was withdrawn voluntarily in November 2022. These changes reflect the ongoing discussions about accelerated approval of novel drugs by FDA taking into account updated and extended trial result as well as results from competing trials in the field ultimately leading to meaningful adaptations in some instances (23, 24).

In Europe, EMA restricts the use of pembrolizumab and atezolizumab in first line mUC to cisplatin-ineligible patients with PD-L1 overexpression (CPS ≥10% in case of pembrolizumab and tumor expression PD-L1 of ≥5% for atezolizumab). Adjuvant nivolumab has been approved by FDA for patients with muscle-invasive urothelial carcinoma at high risk of relapse without PD-L1 restrictions whereas EMA so far limits the use to patients with tumor cell PD-L1 expression of ≥1%. Whether this distinction is still justified appears questionable in view of the discussed lack of evidence of a predictive role of PD-L1 in urothelial cancer. Moreover, updated longterm results of Checkmate 274 indicate possible overall survival benefit in the ITT population (25) leading to further questions about the meaningfulness of PD-L1 expression restrictions.

In conclusion, PD-L1 expression does not appear to be a valuable predictive biomarker in urothelial carcinoma, neither in the muscle-invasive nor in the metastatic disease setting. While subgroup analyses remain hypothesis generating, the totality of data must be taken into account. In our view there are simply too many inconsistencies using PD-L1 immunhistochemistry expression for decision making. Finally, the research community should learn from the mistakes made and aim to develop predictive biomarkers that rely on consistent sampling and measurements with comparable and reproducible assays.

## Patient summary

Regulatory bodies restrict the use of nivolumab (adjuvant setting) and pembrolizumab and atezolizumab (metastatic setting) based on PD-L1 expression in the tumor. This practice appears outdated in view of a review of the full data from the registration trials.
